# Lutein inhibits glutamate-induced apoptosis in HT22 cells via the Nrf2/HO-1 signaling pathway

**DOI:** 10.3389/fnins.2024.1432969

**Published:** 2024-08-13

**Authors:** Zhenhua Li, Zhuohua Cao, Fangmei Chen, Bin Li, Hanyong Jin

**Affiliations:** ^1^Key Laboratory of Natural Medicines of the Changbai Mountain, Ministry of Education, College of Pharmacy, Yanbian University, Yanji, Jilin, China; ^2^Institute of Science and Technology Information Research of Tibet Autonomous Region, Lhasa, China; ^3^Key Laboratory of Pharmaceutical Research for Metabolic Diseases, Department of Pharmacy, Qingdao University of Science and Technology, Qingdao, China; ^4^Department of Medicament, College of Medicine, Tibet University, Lhasa, China

**Keywords:** lutein, glutamate, neuroprotective effects, oxidative stress, apoptosis

## Abstract

**Introduction:**

Excessive glutamate levels induce oxidative stress, resulting in neuronal damage, and cell death. While natural antioxidants show promise for neuroprotection, their effectiveness in the central nervous system (CNS) is limited by the blood -brain barrier. Lutein, a neuroprotective carotenoid, has gained attention for its ability to traverse this barrier and accumulate in various brain regions. This study aimed to elucidate the mechanisms underlying the protective effects of lutein against glutamateinduced cell death in HT22 cells.

**Methods:**

HT22 cells were treated with lutein (1.25-20 μM) for 24 hours. Cell viability, ROS levels, apoptosis, and mitochondrial membrane potential were assessed following lutein pretreatment and glutamate exposure. Protein expression of apoptotic markers was analyzed using Western blotting.

**Results:**

Lutein effectively attenuated glutamate-induced apoptosis due to its antioxidant properties. Additionally, lutein inhibited glutamate-induced mitochondrial-mediated apoptosis. We observed that lutein modulated the nuclear translocation of nuclear factor erythroid 2 -related factor 2 (Nrf2) and upregulated the expression of heme oxygenase-1 (HO-1). Inhibition of HO-1 by tin protoporphyrin (SnPP), a synthetic inhibitor, weakened the protective effect of lutein. Furthermore, we demonstrated that lutein prevented the aberrant activation of MAPKs induced by glutamate, including ERK1/2, p38, and JNK, thereby conferring oxidative protection.

**Discussion:**

Our study highlights the potent antioxidant properties of lutein, which effectively safeguards against glutamate-induced mitochondrial apoptotic cell death through the Nrf2/HO-1 signaling pathway and inhibition of MAPK activation. These findings demonstrate that lutein exerts a neuroprotective effect against glutamate-induced neuronal cell damage.

## 1 Introduction

Glutamate, a crucial endogenous excitatory neurotransmitter in the central nervous system, mediates synaptic transmission through the activation of both metabotropic and ionotropic glutamate receptors (iGluRs) on neurons. It regulates various physiological functions in the cerebral cortex and hippocampus, significantly influencing synaptic plasticity, cognition, learning, and memory (Haroon et al., 2017). However, elevated extracellular glutamate concentrations can induce neuronal cell death through excitotoxicity (Bano et al., 2005), thereby contributing to the pathogenesis of neurodegenerative diseases such as Alzheimer’s, Parkinson’s, and amyotrophic lateral sclerosis (Barnham et al., 2004), as well as the onset and progression of mental illnesses like depression and anxiety disorders (Salim, 2014; Black et al., 2015). Many studies have described glutamate-induced cytotoxicity occurring through both receptor-mediated and non-receptor-mediated pathways. iGluRs are classified into four subtypes based on their ligand binding properties and sequence similarity, including α-amino-3-hydroxy-5-methyl-4-isoxazolepropionic acid (AMPA), kainate, N-methyl-d-aspartate (NMDA), and delta receptors (Collingridge et al., 2009), which predominantly mediate most of the fast excitatory neurotransmission in the brain. In non-receptor-mediated cytotoxicity, excess extracellular glutamate inhibits the neuronal glutamate/cystine antiporter (Murphy et al., 1989). This inhibition results in decreased cystine uptake, leading to reduced levels of glutathione, impaired clearance of reactive oxygen species (ROS), and the subsequent accumulation of these species, causing oxidative stress toxicity (Murphy et al., 1989). HT22 neuronal cells are advantageous for studying non-receptor-mediated cytotoxicity and oxidative stress responses induced by glutamate due to their lack of ionotropic glutamate receptors (Murphy et al., 1989; Maher and Davis, 1996).

The dynamic levels of ROS within mitochondria are closely associated with essential cellular functions. However, the disruption of mitochondrial redox balance in cells from various tissues can lead to pathological developments (Sies and Jones, 2020).This impairment compromises cellular function and disrupts the organism’s normal physiological activities, ultimately inducing oxidative stress. Consequently, lipid peroxidation, cellular damage, and the opening of mitochondrial permeability transition pores (MPTP) are triggered, thereby facilitating apoptosis in various tissue cells (Sies and Jones, 2020). Neuronal cells in the central nervous system, characterized by their heightened metabolism, lipid richness, and lower antioxidant levels, are notably prone to oxidative stress damage compared to other tissue cells (Floyd and Carney, 1992). Oxidative stress, whether induced by an overproduction of mitochondrial ROS or impairment of antioxidant defenses, results in mitochondrial dysfunction and initiates the cellular death cascade (Islam, 2017). Mitochondrial dysfunction-induced cell death typically begins with mitochondrial outer membrane permeabilization (MOMP), a process activated by pro-apoptotic effectors, Bax and Bak. The loss of membrane integrity results in the release of cytochrome c into the cytosol, where it forms a complex known as the apoptosome along with the proapoptotic cytosolic factor *APAF1*. This apoptosome complex then activates caspase 9, which subsequently cleaves caspases 3 and 7 leading to rapid cellular demolition (Bock and Tait, 2020).

Scavenging ROS through Nrf2-mediated induction of antioxidant enzymes is essential for maintaining cellular redox homeostasis (Loboda et al., 2016). Heme oxygenase-1 (HO-1) is a major target gene regulated by Nrf2, playing a critical role in cellular protection against harmful stimuli from both endogenous and exogenous sources (Winyard et al., 2005). Its antioxidant function involves preventing free heme from engaging in oxidative reactions and, along with its enzymatic products biliverdin and carbon monoxide (CO), conferring antioxidant, anti-inflammatory, anti-apoptotic, and vasodilatory effects, as well as enhancing tissue microcirculation. Under normal conditions, Nrf2 is bound to its cytoplasmic inhibitor Keap1. Upon external stimulation, Nrf2 dissociates, translocates to the nucleus, and binds to the HO-1 promoter antioxidant response element (ARE), thereby activating HO-1 gene expression and providing cellular protection. Previous studies have shown that the Nrf2/HO-1 signaling pathway prevents glutamate-induced oxidative stress in HT22 cell death (Wang et al., 2016; Huang et al., 2018; Cuadrado et al., 2019; Song et al., 2019b; Gao et al., 2023; Wang et al., 2024).

Intracellular oxidative stress can also be induced by the mitogen-activated protein kinase (MAPK) signaling pathway, leading to the production of reactive oxygen species (ROS) (Son et al., 2011). The MAPK protein belongs to the serine/threonine protein kinase family and plays an important role in the expression of various proteins involved in cell differentiation, inflammation, and apoptosis (Yue and López, 2020). MAPKs, such as ERK1/2, p38, and JNK, serve as key mediators in converting extracellular signals into a multitude of cellular responses, encompassing cell growth, migration, proliferation, differentiation, and apoptosis (Yue and López, 2020). Several studies have demonstrated that oxidative stress resulting from glutamate exposure can activate MAPKs, leading to hippocampal neuronal apoptosis (Fukui et al., 2009; Ortuño-Sahagún et al., 2014; Park et al., 2019; Song et al., 2019a,b; Baek et al., 2021).

Lutein has beneficial effects in delaying the progression of age-related eye conditions and shows promise in protecting against neurodegenerative diseases by enhancing antioxidant enzyme activity in the brain (Ahn and Kim, 2021). Several studies have reported that lutein exhibits protective effects against neuronal damage caused by glutamate, enhancing antioxidant enzyme activity and reducing inflammation in both neuroblastoma and microglial cells (Pap et al., 2022; Phoraksa et al., 2023). However, further research is necessary to fully comprehend its effectiveness against glutamate-induced excitotoxicity, particularly concerning metabotropic glutamate receptors. In this study, we demonstrated that lutein prevents glutamate-induced apoptosis in HT22 cells by reducing oxidative stress and mitochondrial damage. This protective effect is mediated through the Nrf2/HO-1 and MAPKs pathways, suggesting significant neuroprotective potential.

## 2 Materials and methods

### 2.1 Reagents

Lutein (purity of >98%) was purchased from Chengdu Must Bio-technology Co., Ltd. (Chengdu, China). All chemicals used in this study were purchased from Gibco BRL Co. (Grand Island, NY, United States). The following kits were purchased: Reactive Oxygen Species Assay Kit, Mitochondrial Membrane Potential Assay Kit with JC-1, and One Step of TUNEL Apoptosis Assay Kit were purchased from Beyotime Biotechnology Co., Ltd. (Shanghai, China). MTT [3-(4,5-Dimethylthiazol-2-yl)-2,5-diphenyltetrazolium bromide] were purchased from Beijing Solarbio Science & Technology Co., Ltd. (Beijing, China). Cobalt protoporphyrin (CoPP) and tin protoporphyrin (SnPP) were obtained from Sigma-Aldrich Life Science & Technology Co., Ltd. (St. Louis, MO, United States).

### 2.2 Cell culture

Mouse hippocampal HT22 cells were obtained from the Chinese Type Culture Collection Center (Wuhan, China) and were cultured in DMEM medium containing 10% heat-inactivated FBS (BDBIO, Hangzhou, China) at 37°C in an incubator with 5% carbon dioxide.

### 2.3 Cell viability assay

Cell viability was evaluated by a 3-[4,5-Dimethylthiazol-2-yl]-2,5-diphenyltetrazolium bromide (MTT) assay. Cells (8 × 10^3^ cells/well in 96-well plates) were incubated with MTT at a final concentration of 0.5 mg/mL for 4 h Dimethyl sulfoxide (DMSO) (Sigma-Aldrich) was added to dissolve dark blue formazan crystals formed in the viable cells. Optical density was measured at 490 nm using a microplate reader (Thermo Multiskan Sky, Waltham, MA, United States). The optical density of formazan formed in the control (untreated) cells was considered as 100% cell viability.

### 2.4 Analyses of ROS levels in cells

According to the directions provided by the manufacturer, the intracellular ROS level was assayed with the fluorescent probe 2,7-dichlorodi-hydrofluorescein diacetate (DCF-DA; Solarbio, Beijing, China). HT22 cells (1.0 × 10^4^ cells/well) were cultured in 24-well plates. After 20 min of incubation at 37°C with a diluted DCFH-DA probe, serum-free medium was used to wash the cells three times. The cells were then collected after being washed three times with PBS. With the use of a spectrofluorometer (Spectramax Gemini XS; Molecular Devices, Sunnyvale, San Jose, CA, United States), the fluorescence intensity was detected at wavelengths of 530 nm for the emission and 484 nm for the excitation.

### 2.5 Mitochondrial membrane potential assay

Changes in mitochondrial membrane potential in HT22 cells were measured using the mitochondrial membrane potential assay kit with JC-1 (Beyotime, Shanghai, China). After being cultivated in a 6-well plate and treated with glutamate and/or lutein, the cells were washed with PBS. The JC-1 staining solution was added to each well and incubated for 30 min at 37°C. Mitochondria were visualized using a fluorescence microscope (Provis AX70, Olympus Optical Co., Tokyo, Japan) after cells were rinsed with JC-1 staining solution (1 ×). Green fluorescence indicates mitochondrial depolarization, while red fluorescence indicates normal mitochondria. The red/green fluorescence intensity ratio is utilized to assess mitochondrial depolarization.

### 2.6 Cell apoptosis assay

One step of TUNEL Apoptosis Assay Kit (Beyotime, Shanghai, China) was used to investigate the apoptosis of HT22 cells. Briefly, cells were treated with 0.3% Triton X-100 and the TUNEL detection solution at 37°C in the absence of light after being fixed in 4% paraformaldehyde for 60 min. Subsequently, the nuclei were stained with DAPI staining solution, sealed with a microscope cover glass, and observed under a fluorescence microscope (Olympus Optical Co.) after being washed three times with PBS buffer solution.

### 2.7 Western blotting

The samples (40 μg protein) were electrophoresed by 10% SDS-polyacrylamide gel electrophoresis (SDS-PAGE) and transferred to a nitrocellulose membrane (Pall Corporation, NY, United States). The membrane was sealed for 2 h in TBST with 5% nonfat dry milk. The primary antibodies, including p-JNK antibody (Cat: #AF3318, 1:1000), JNK antibody (Cat: #AF6318, 1:1000), p-ERK antibody (Cat: #AF1015, 1:1000), ERK antibody (Cat: #AF0155, 1:1000), p-p38 antibody (Cat: #AF6456, 1:1000), p-p38 antibody (Cat: #AF4001, 1:1000), HO-1 antibody (Cat: #AF5393, 1:1000), Nrf2 antibody (Cat: #AF0639, 1:1000), caspase 3 antibody (Cat: #AF6311, 1:1000), caspase 9 antibody (Cat: #AF6348, 1:1000), cleaved caspase 3 antibody (Cat: #AF7022, 1:1000), cleaved caspase 9 antibody (Cat: #AF5240, 1:1000), β-Actin antibody (Cat: #AF7018, 1:1000), Lamin B1 antibody (Cat: #AF5161, 1:1000), and HRP-conjugated Affifinipure Goat Anti-Rabbit IgG (H + L) secondary antibodies (#S0001, 1:10000) were obtained from Affinity Biosciences (OH, United States). An Ultra High Sensitivity ECL Kit (MCE, State of New Jersey, United States) and ChemiDoc image analyzer (Tanon 4,600, Tanon, China) are used to display protein imprinting. Finally, protein quantitative analysis was used by an ImageJ analysis program (National Institutes of Health, United States).

### 2.8 Subcellular fractionation

HT22 cells were lysed with a mixture of RIPA lysis buffer [50 mM Tris (pH 7.4), 150 mM NaCl, 1% Triton X-100, 1% sodium deoxycholate, 0.1% SDS, and general protease and phosphatase inhibitors] (MCE, State of New Jersey, United States) and 1 mM phenylmethylsulfonyl fluoride (PMSF; Solarbio, Beijing, China) for 10 min at 4°C, and the supernatant was collected by centrifugation to extract total protein. The extraction of nuclear and cytosolic proteins was performed using the Nuclear Protein Extraction Kit (Solarbio, Beijing, China) from HT22 cells. Bradford Protein Assay Kit (Solarbio, Beijing, China) was used to measure the protein extracts.

### 2.9 Nrf2 immunofluorescence

For the nuclear translocation of Nrf2, immunofluorescence experiments were performed. HT22 cells were permeabilized with 0.5% Triton X-100 after being treated with lutein (10 μM) and fixed with 4% paraformaldehyde. After blocking non-specific binding sites with 1% FBS for an hour, cells were incubated with a Nrf2 antibody (1,200) at 4°C for an overnight period and were incubated with the secondary conjugated antibodies (Alexa Fluor 488; Invitrogen, Carlsbad, CA, United States) at 4°C for 10 min. In order to see the nuclei, DAPI (1 μg/mL) labeling was completed. A Provis AX70 fluorescent microscope (Olympus Optical, Tokyo, Japan) was used to view and capture stained cells.

### 2.10 Statistical analysis

All data described in this study were replicated at least three times and are presented as the mean ± standard error of mean (S.E.M). Statistical analyses were conducted using GraphPad Prism 7 software (San Diego, CA, United States), and group differences were assessed using one-way ANOVA. A significance level of *p* < 0.05 was considered as indicating statistically significant.

## 3 Results

### 3.1 Effect of lutein on cell viability in glutamate-induced cytotoxicity

**Figure 1:**
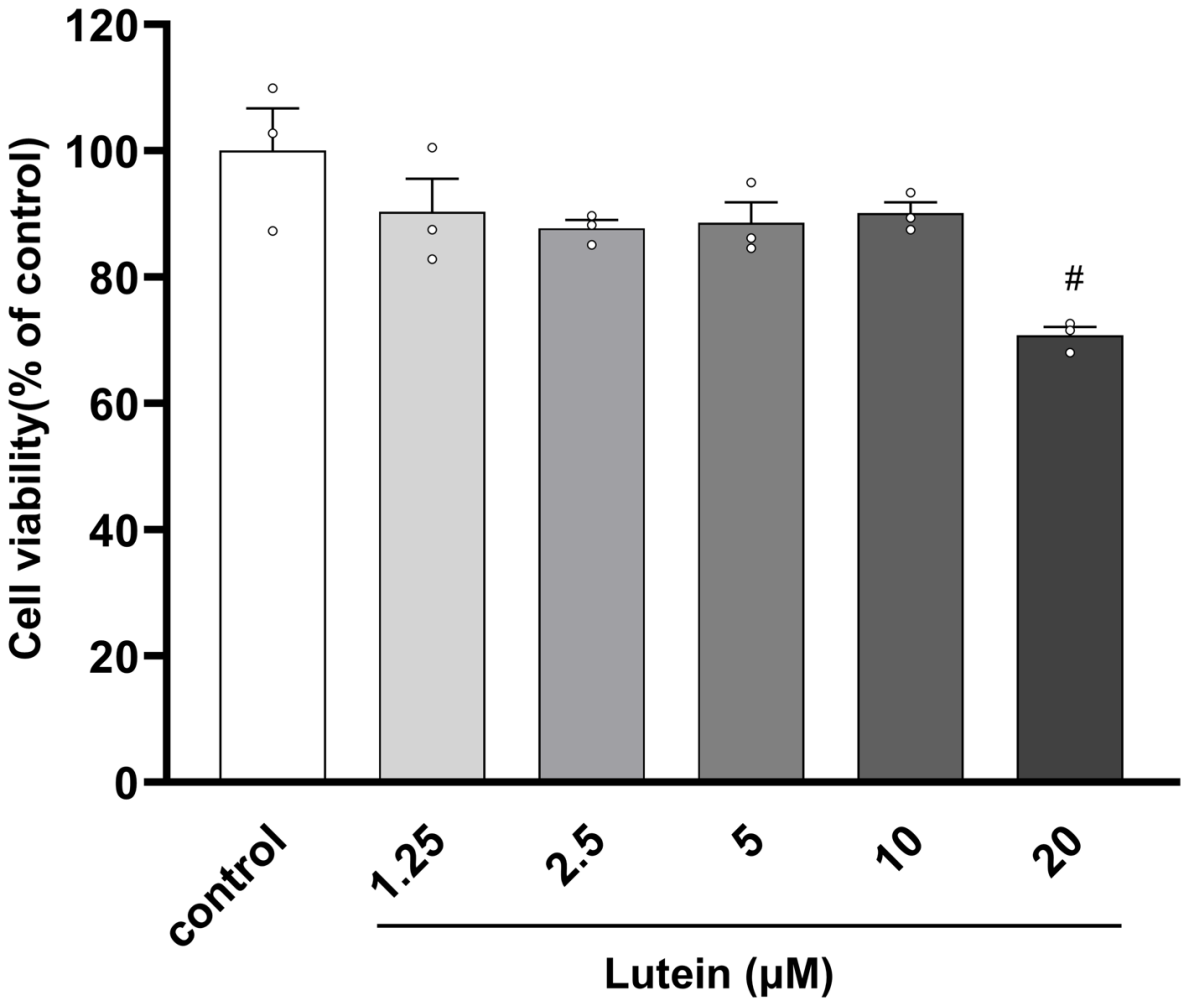
Effect of Lutein on the viability of HT22 cells. HT22 cells were incubated with various concentrations of Lutein for 24 h, and cell viability was evaluated. Data are means ± S.E.M of three independent experiments. Statistical significance is denoted as follows: ^#^*p* < 0.05, vs. control group.

**Figure 2:**
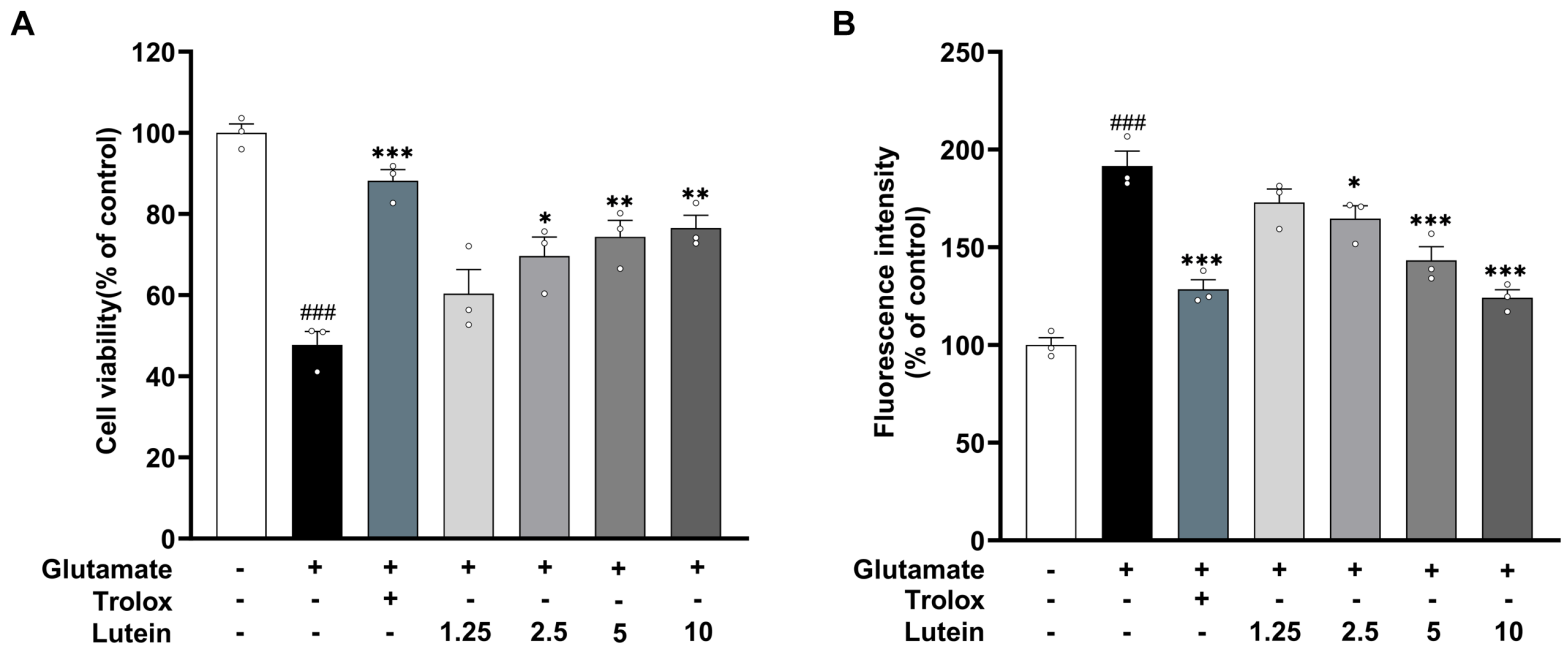
Neuroprotective effects of Lutein on glutamate-induced toxicity in HT22 cells. (**A,B**) HT22 cells were stimulated with glutamate (20 mM) for 24 h after pretreatment with an increasing concentrations of Lutein or Trolox (50 μM) for 12 h. Cell viability **(A)** and intracellular ROS levels **(B)** were measured. Data are means ± S.E.M of three independent experiments. Statistical significance is denoted as follows: ^###^*p* < 0.001 vs. control group; ^*^p < 0.05, ^**^p < 0.01, ^***^p < 0.001 vs. glutamate treated group.

To assess the impact of lutein on HT22 cells, cell viability was determined using the MTT assay after incubation with various concentrations of lutein (1.25, 2.5, 5, 10, 20 μM) for 24 h (Figure 1). The results indicated that lutein did not induce cytotoxicity in HT22 cells except at the highest concentration (20 μM) (Figure 1). Consequently, the maximum concentration of lutein was restricted to 10 μM in all subsequent experiments. Next, we evaluated the ability of lutein to counteract glutamate-induced cytotoxicity in HT22 cells. To determine the neuroprotective effect of lutein against glutamate-induced cytotoxicity in HT22 cells, the cells were treated with 20 mM glutamate with or without varying concentrations of lutein (1.25, 2.5, 5, and 10 μM) for 24 h (Figure 2A). Treatment with 20 mM glutamate significantly reduced the cell viability of HT22 neuronal cells compared to the control, which was reversed by pretreatment with 10 μM of Lutein (Figure 2A). Consequently, it was observed that lutein significantly inhibited HT22 cell death induced by glutamate at a level similar to that of the positive control, Trolox. Consistent with this findings, quantitative fluorescence intensity results demonstrated that glutamate exposure elevated increased intracellular ROS levels, a phenomenon significantly attenuated by pretreatment with 10 μM lutein and Trolox (Figure 2B). These findings suggest that the antioxidant properties of lutein may mitigate oxidative stress-mediated neuronal cell death induced by glutamate.

### 3.2 Effects of lutein against glutamate-induced mitochondrial apoptotic death in HT22 cells

**Figure 3:**
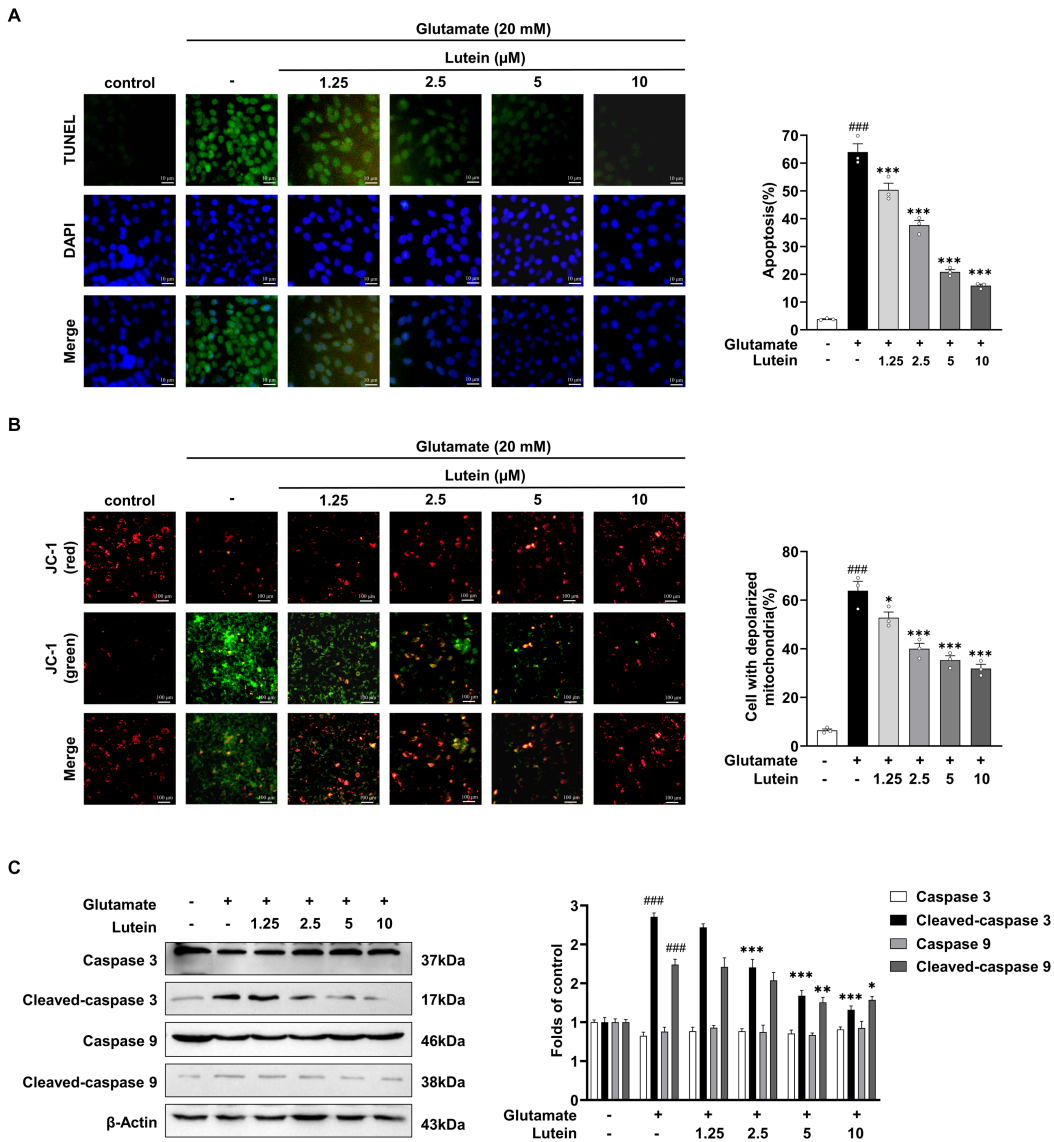
Lutein participates in early cell apoptosis by inhibiting glutamate mediated mitochondrial membrane potential destruction. **(A)** HT22 cells were exposed to 20 mM glutamate with varying concentrations of lutein (1.25, 2.5, 5, and 10 μM) and subjected to the FITC test (one-step TUNEL apoptosis test kit) to assess the number of apoptotic and dead cells, respectively. The bar graph indicates the percentage of apoptotic cells. Data are presented as the mean value ± S.E.M of three independent experiments. **(B)** Representative images of JC-1 staining were detected using fluorescence microscopy. The bar graph indicates the percentage of cells exhibiting mitochondrial depolarization. Data are presented as the mean value ± S.E.M of three independent experiments. **(C)** Western blot analysis was conducted. β-Actin serving as a loading control. The bars represent the fold-increase in the levels of cleaved caspase 3 and cleaved caspase 9 compared to control cells. Data are presented as the mean value ± S.E.M of three independent experiments. Statistical significance is denoted as follows: ^###^*p* < 0.001 vs. control cells; ^*^p < 0.05, ^**^p < 0.01, ^***^p < 0.001 vs. glutamate treated group.

Previous studies have indicated that glutamate-induced cell death in HT22 cells primarily proceeds through the apoptotic pathway (Kritis et al., 2015; Huang et al., 2018; Song et al., 2019a; Gao et al., 2023). In this study, we aimed to investigate the impact of lutein on glutamate-induced apoptotic cell death. HT22 cells were treated with increasing concentrations of lutein in the presence of glutamate to assess its effects. DAPI staining demonstrated a reduction in glutamate-induced nuclear condensation following lutein treatment (Figure 3A). Additionally, the number of FITC-positive HT22 cells induced by glutamate decreased significantly with increasing doses of lutein (Figure 3A). Glutamate exposure led to elevated intracellular ROS levels, contributing to neuronal cell death through oxidative stress mechanisms (Figures 2A,B). To explore lutein’s potential antiapoptotic mechanisms, we conducted a JC-1 staining assay to evaluate its ability to counteract glutamate-induced apoptosis by preventing mitochondrial dysfunction triggered by oxidative stress. Glutamate markedly induced mitochondrial membrane potential depolarization in HT22 cells (Figure 3B). However, pretreatment with lutein dose-dependently attenuated glutamate-induced MOMP depolarization (Figure 3B). Additionally, immunoblot analysis revealed the activation of caspase 9 and caspase 3 due to glutamate-induced disruption of mitochondrial membranes (Figure 3C). Conversely, lutein treatment dose-dependently inhibited apoptosis induction by preventing the formation of cleaved caspase 9 and caspase 3 (Figure 3C). These findings indicate that lutein exerts anti-apoptotic effects by inhibiting glutamate-induced mitochondrial apoptotic death in HT22 cells.

### 3.3 Protective effects of lutein on glutamate-induced oxidative stress

**Figure 4:**
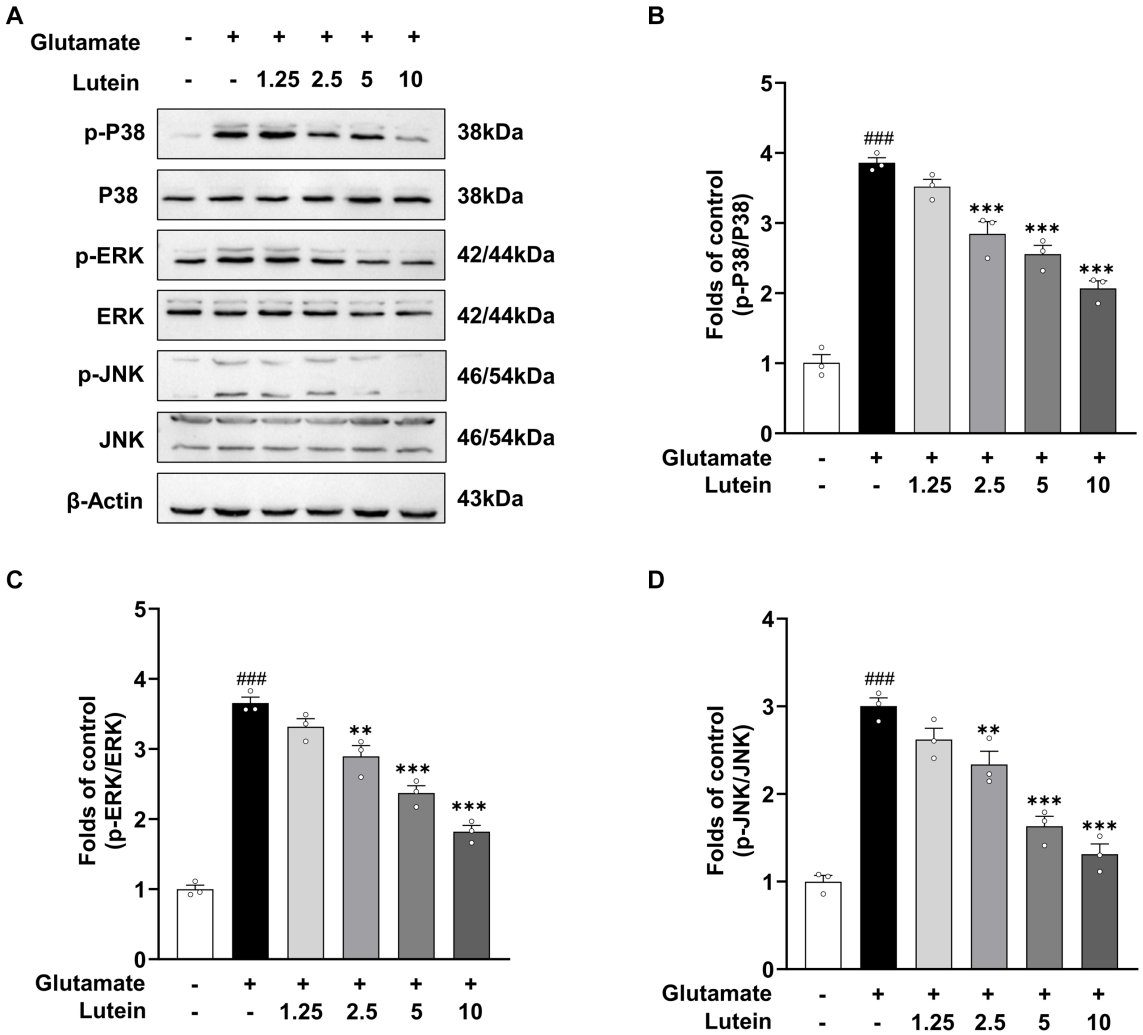
Lutein inhibits glutamate-induced MAPK activation in HT22 cells. **(A)** HT22 cells were exposed to 20 mM glutamate in the presence or absence of indicated concentrations for 8 h. Western blot analysis was performed using indicated antibodies, with β-Actin serving as a loading control. **(B–D)** The bars represent the fold-increases of P38, ERK, and JNK phosphorylation levels compared to the control cells. Data are presented as the mean value ± S.E.M of three independent experiments. Statistical significance is denoted as follows: ^###^*p* < 0 0.001 vs. control group; ^**^p < 0.01, ^***^p < 0.001 vs. glutamate treated group.

Previous studies have demonstrated that glutamate-induced oxidative stress can trigger aberrant activation of MAPK pathways, leading to neuronal cell death (Xia et al., 1995; Horstmann et al., 1998; Stanciu et al., 2000; Szydlowska et al., 2010). To investigate whether lutein can inhibit the aberrant activation of MAPKs by glutamate, we treated HT22 neuronal cells with increasing concentrations of lutein in the presence or absence of glutamate and conducted immunoblotting analysis (Figure 4A). The results showed that the activation of MAPKs, including the phosphorylation of ERK, p38, and JNK induced by glutamate, was significantly reduced in a dose–response manner upon treatment with increasing concentrations of lutein (Figures 4A–D). These findings suggest that the inhibition of MAPKs is a molecular mechanism underlying lutein-mediated neuroprotection against glutamate-induced cell death in HT22 cells.

### 3.4 Antioxidant properties of lutein on HT22 cells by regulating Nrf2/HO-1 axis

**Figure 5:**
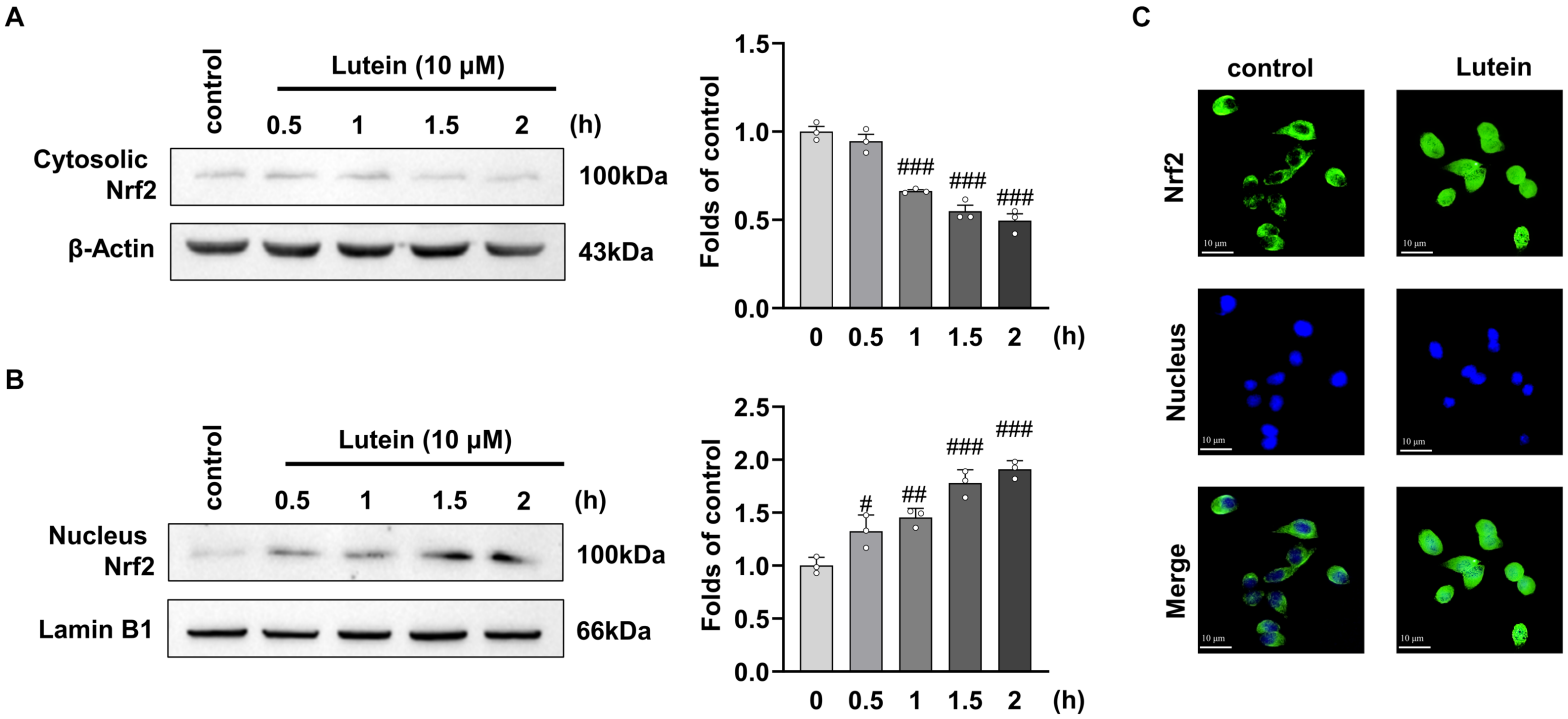
Lutein increases Nrf2 nuclear translocation in HT22 cells. (**A,B**) Translocation of cytosolic Nrf2 into the nucleus was assessed in HT22 cells incubated with 10 μM Lutein for indicated times. The cytosolic and nucleus fractions were isolated and analyzed by western blotting with β-Actin and Lamin B1, respectively. Bars indicate fold-increases compared to the control cells. Data are mean ± S.E.M of three independent experiments. Statistical significance is denoted as follows: ^#^*p* < 0.05, ^##^*p* < 0.01, ^###^*p* < 0.001 vs. control cells. (**C**) Representative immunofluorescence of Nrf2 expression in HT22 cells is shown. Representative immunofluorescence of Nrf2 expression in HT22 cells is shown. Staining of Nrf2 (green labeled) and the nucleus (blue labeled) was detected using fluorescent microscopy.

The Nrf2/HO-1 signaling pathway has been implicated in the progression of various neurological complications, including glutamate-induced oxidative cell death (Sacks et al., 2018). Previous studies have suggested that lutein activates Nrf2, leading to the expression of antioxidant genes such as HO-1. To investigate the potential of lutein as a modulator of the Nrf2/HO-1 signaling pathway in different neurodegenerative disorders, we examined its impact on Nrf2 translocation and HO-1 expression in HT22 cells. Immunofluorescence and immunoblot analysis revealed a significant increase in Nrf2 accumulation within the nucleus after a 2 h treatment with 10 μM lutein, compared to the control group (Figures 5A–C). Conversely, the levels of cytosolic Nrf2 were notably decreased (Figure 5A), indicating that lutein effectively enhanced the translocation of Nrf2 to the nucleus in HT22 neuronal cells, leading to the activation of Nrf2 downstream target gene expression.

**Figure 6:**
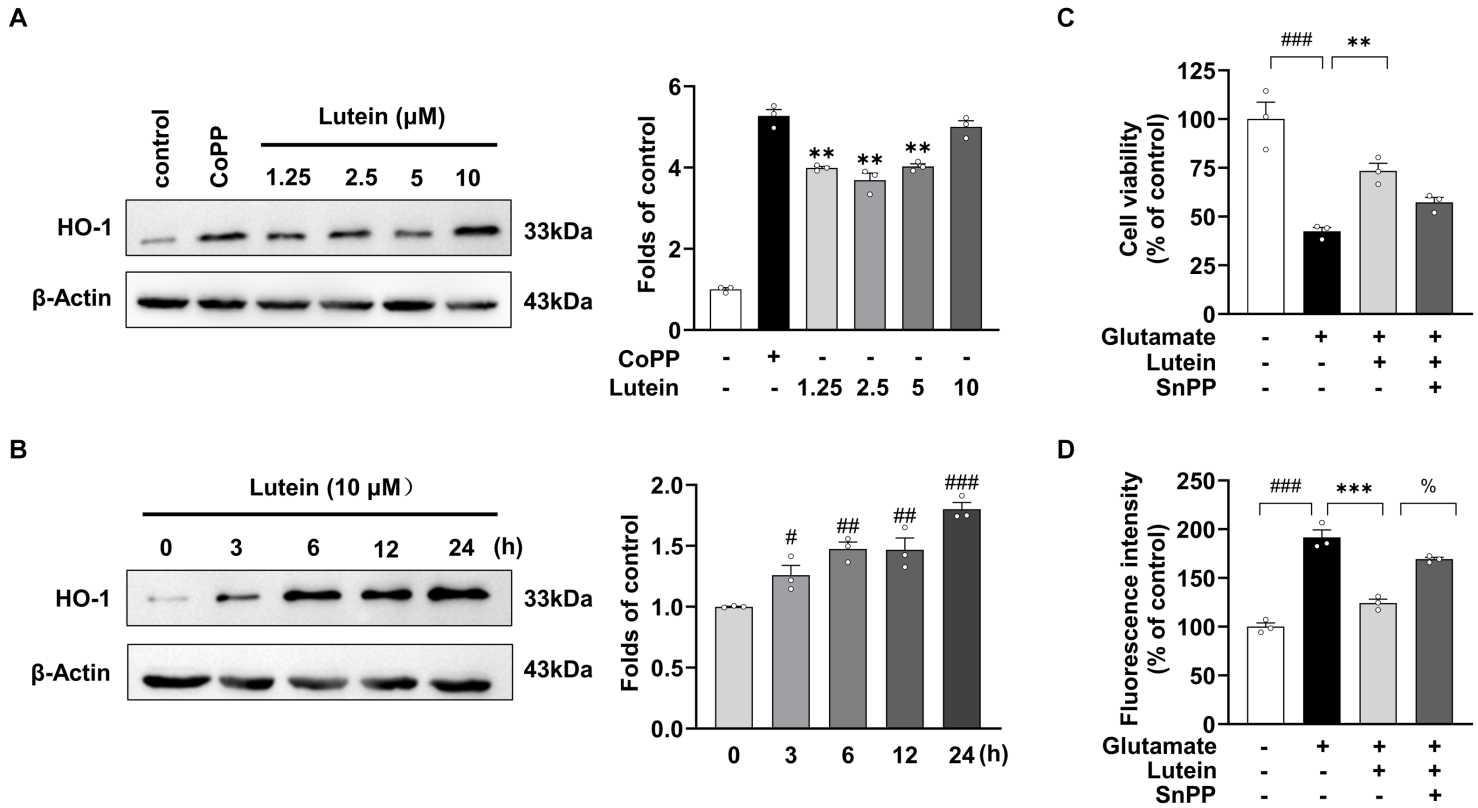
Effects of Lutein on HO-1 expression in HT22 cells. (**A**) HO-1 expression was evaluated in the presence of varying concentrations of Lutein and compared to 20 μM CoPP after 12 h. Western blot analysis was conducted with β-Actin as the control. Bars represent fold-increases in HO-1 expression. Data are presented as the mean value ± S.E.M of three independent experiments. Statistical significance is denoted as follows: ^**^p < 0.001 vs. CoPP treated group. (**B**) Western blot analysis was performed after incubation with 10 μM Lutein for indicated times, with β-Actin as a loading control. Data are presented as the mean value ± S.E.M of three independent experiments. Statistical significance is denoted as follows: ^#^*p* < 0.05, ^##^*p* < 0.01, ^###^*p* < 0.001 vs. CoPP treated cells group. (**C,D**) HT22 cells were treated with 20 mM glutamate and subsequently incubated with either 50 μM SnPP or 10 μM Lutein, followed by the assessment of cell viability (**C**) and intracellular ROS levels (**D**). Data are presented as the mean value ± S.E.M of three independent experiments. Statistical significance is denoted as follows: ^%^*p* < 0.05, ^***^p < 0.001, ^###^*p* < 0.001 vs. corresponding group.

Given the demonstrated role of Nrf2-driven antioxidant gene expression of HO-1 in protecting against glutamate-induced oxidative damage in HT22 neuronal cells (Kim et al., 2012), we aimed to investigate the effects of lutein on HO-1 expression in HT22 neuronal cells. Immunoblot analysis showed that HO-1 expression significantly increased with lutein treatment in a dose- and time-dependent manner (Figures 6A,B). Cobalt protoporphyrin (CoPP), an HO-1 inducer, also significantly increased HO-1 expression, comparable to the level induced by 10 μM lutein (Figure 6A). Next, we further elucidated whether the increase in Nrf2 nuclear translocation and HO-1 expression by lutein contributed to the protection of HT22 neuronal cells from glutamate-induced oxidative cell death. To validate the role of HO-1, we utilized tin protoporphyrin (SnPP), a synthetic inhibitor of HO-1, to inhibit its activity. In Figure 6C, we observed that the presence of 50 μM SnPP partially blocked the protective effect of lutein against glutamate-induced HT22 cell death. Consistently, treatment with SnPP also partially prevented the reduction of intracellular ROS levels induced by lutein in HT22 neuronal cells (Figure 6D). These findings suggest that lutein modulation of Nrf2-mediated expression of HO-1 could be a potential molecular mechanism for eliminating glutamate-induced oxidative stress by lutein, thereby partially inhibiting glutamate-induced oxidative cell death.

## 4 Discussion

Naturally occurring antioxidants have garnered significant attention due to their neuroprotective effects against oxidative stress (Pérez-Torres et al., 2021). However, their efficacy in the central nervous system (CNS) is often limited by the blood–brain barrier. Lutein, capable of crossing this barrier and accumulating in various brain regions, significant accumulation has been observed in the hippocampal regions of both humans and non-human primates, presents a promising candidate for neuroprotection against degenerative diseases (Lieblein-Boff et al., 2015; Mohn et al., 2017; Iyer et al., 2024). Although extensively studied, the specific neuroprotective effects of lutein in hippocampal neurons had not been investigated prior to our research. HT22 hippocampal neuron cells, selected for their resistance to glutamate receptor-mediated excitotoxicity, are commonly used as an *in vitro* model to investigate the neuroprotective properties of natural compounds against oxidative stress induced by non-receptor glutamate pathways. Glutamate-induced oxidative stress in neuronal cells results from altered cystine/glutamate antiporter activity and other pathological factors such as ischemia and trauma (Newcomb et al., 1997).

Lutein, a naturally occurring carotenoid obtained through dietary intake (Berendschot et al., 2000), has shown significant neuroprotective effects in both clinical trials and experimental studies (Yuan et al., 2021; Agarwal et al., 2022). It accumulates in critical areas like the retina and the CNS, offering protection against oxidative stress-induced damage, which is particularly relevant in neurodegenerative disorders such as Alzheimer’s and Parkinson’s diseases (Feart et al., 2016; Mullan et al., 2017). The primary mechanism underlying lutein’s neuroprotection is its antioxidant properties (Iyer et al., 2024). Although studies have shown lutein’s ability to mitigate ROS generation induced by various factors in CNS cells, such as b.END.3, PC12, SH-SY5Y, and BV-2 cells (Liu et al., 2017; Singhrang et al., 2018; Pap et al., 2021), research has primarily focused on its role in countering oxidative stress in neuronal cells triggered by amyloid deposition and external oxidants. Excitotoxicity, induced by glutamate, is recognized as a crucial factor in the development and progression of neurodegenerative diseases. Addressing glutamate-induced excitotoxicity and oxidative stress with safe and effective natural compounds is thus crucial in treating such conditions. This study aims to enrich our understanding of lutein’s pharmacological properties, thereby broadening our comprehension of its specific mechanisms underlying therapeutic potential for neurodegenerative diseases. Therefore, in this study, we constructed an *in vitro* glutamate-induced HT22 cell injury model to explore the neuroprotective mechanism of lutein in mouse hippocampal neuronal cell lines. We found that lutein can decrease the cytotoxicity of glutamate-induced HT22 cells in a concentration-dependent manner (Figure 2A), depends on its antioxidant properties (Figure 2B).

Excessive extracellular glutamate levels can trigger neuronal death through excitotoxicity, ferroptosis, and mitochondrial dysfunction, all of which disrupt intracellular redox balance (Vaglio-Garro et al., 2024). Our study, consistent with prior research, highlights the association between neuronal apoptosis and oxidative stress-induced accumulation of ROS within mitochondria, leading to mitochondrial damage and caspase-mediated apoptosis (Figure 3). In our investigation, lutein demonstrated notable protective effects against glutamate-induced MOMP depolarization in HT22 cells in a dose-dependent manner (Figure 3B). Moreover, lutein effectively inhibited the activation of caspase 3 and caspase 9, thereby mitigating apoptosis (Figure 3C). The precise mechanism underlying glutamate-induced cell death in HT22 cells remains debated, with evidence suggesting alternative pathways at lower concentrations. For instance, lower glutamate concentrations (≤ 5 mM) can induce caspase-independent DNA fragmentation (Fukui et al., 2009), while specific concentrations can trigger ferroptosis, a process attenuated by compounds like quercetin through the SIRT1/Nrf2/SLC7A11/GPX4 pathway (Xie et al., 2022). Furthermore, in other cell lines like the human neuroblastoma cell line SH-SY5Y, glutamate treatments induce oxidative stress, inflammation, iron accumulation, and lipid peroxidation. Lutein counteracts these effects by reducing ROS, suppressing pro-inflammatory cytokines, preventing iron accumulation, and downregulating lipid peroxidation-associated gene expression, thus inhibiting ferroptosis (Xie et al., 2022). Our findings suggest that lutein protects against caspase-dependent apoptosis following 20 mM glutamate treatment in HT22 cells. However, further research is needed to explore whether lutein’s protective effects extend to other cell death pathways in HT22 cells.

Mitogen-activated protein kinases (MAPKs) play crucial roles in various cellular processes, including cell proliferation, differentiation, inflammation, and apoptosis. Oxidative stress triggers the activation of MAPK pathways, such as JNK and p38, through apoptosis signal-regulating kinase 1 (ASK1), ultimately inducing apoptosis (Saitoh et al., 1998). Although studies have linked JNK, p38, and ERK activation to glutamate-induced apoptosis in HT22 cells, lutein inhibits MAPK activation under oxidative stress, providing cellular protection. For instance, lutein suppresses ERK1/2, JNK1, and p38 activation in cells exposed to oxidative stress inducers (Hu et al., 2021), and reduces lipid peroxidation and JNK and p38 activation in human lens epithelial cells exposed to UVB radiation (Silván et al., 2016). Similarly, our research shows that lutein inhibits JNK, p38, and ERK activation in HT22 cells under oxidative stress induced by glutamate, thereby reducing apoptosis (Figure 4). Overall, high concentrations of glutamate induce MAPK and caspase activation, leading to apoptotic cell death, which is dependent on mitochondrial dysfunction caused by oxidative stress-induced ROS accumulation.

*Nrf2*, a transcription factor, plays a crucial role in regulating antioxidant stress proteins. Normally, Nrf2 levels are kept low by Keap1, which promotes its degradation via ubiquitination. However, exposure to ROS or electrophilic agents causes Nrf2 to dissociate from Keap1 and translocate to the nucleus. There, it binds to antioxidant response element (ARE) sequences, activating the transcription of various antioxidant enzymes like HO-1, GST, SOD, and NQO-1, which help detoxify ROS (Huang et al., 2002). Lutein has been found to enhance the nuclear translocation of Nrf2, leading to increased expression of Nrf2-targeted antioxidant enzymes and exerting an antioxidant effect. Studies show that lutein activates Nrf2, resulting in elevated expression of protective enzymes like HO-1 and NQO1, which mitigates oxidative stress in microglial cells exposed to lipopolysaccharide (Wu et al., 2015) Additionally, lutein reverses H_2_O_2_-induced down-regulation of HO-1 mRNA in PC12 cells (Hu et al., 2021). Our research confirms these findings, demonstrating that lutein boosts the nuclear translocation of Nrf2 and upregulates HO-1 expression in HT22 cells (Figure 5). The antioxidant properties of lutein may stem from the upregulation of Nrf2-dependent antioxidant genes and its phenolic hydroxyl structure’s inherent reductive capacity against ROS. Moreover, we observed that the HO-1 inhibitor, SnPP, significantly diminishes lutein’s antioxidant efficacy, highlighting the crucial role of HO-1 upregulation in protecting HT22 cells from glutamate-induced oxidative stress (Figure 6).

In undifferentiated HT22 cells devoid of glutamate receptors, we have confirmed that lutein confers protection against non-receptor-mediated glutamate neurotoxicity via its antioxidant properties. Non-receptor-mediated glutamate neurotoxicity is pivotal in inducing neuronal cell toxicity under pathological states, including hypoxia and neuronal injury, characterized by the release and accumulation of intracellular glutamate in the synaptic cleft at elevated concentrations. The *in vivo* hippocampus consists of differentiated neurons with glutamate and cholinergic receptors, operating at lower synaptic cleft glutamate levels, further research is needed to elucidate lutein’s protective effect against NMDA receptor-mediated excitotoxicity. Reports indicate that the antioxidant dithiothreitol (DTT) exhibits a diminished capacity to reverse the decrease in cell viability in differentiated HT22 cells with NMDA receptors treated with glutamate, in comparison to undifferentiated HT22 cells (He et al., 2013). Conversely, another study demonstrated that lutein significantly mitigated the upregulation of Bax, cytochrome c, p-p38 MAPK, and p-c-Jun in the retinal ganglion cells of rats administered NMDA, ameliorating neuronal damage from excitotoxicity (Zhang et al., 2016). Despite the diminished role of oxidative stress in glutamate receptor-mediated excitotoxicity, lutein might still afford protection against neuronal excitotoxicity via mechanisms distinct from its antioxidant activity.

**Figure 7:**
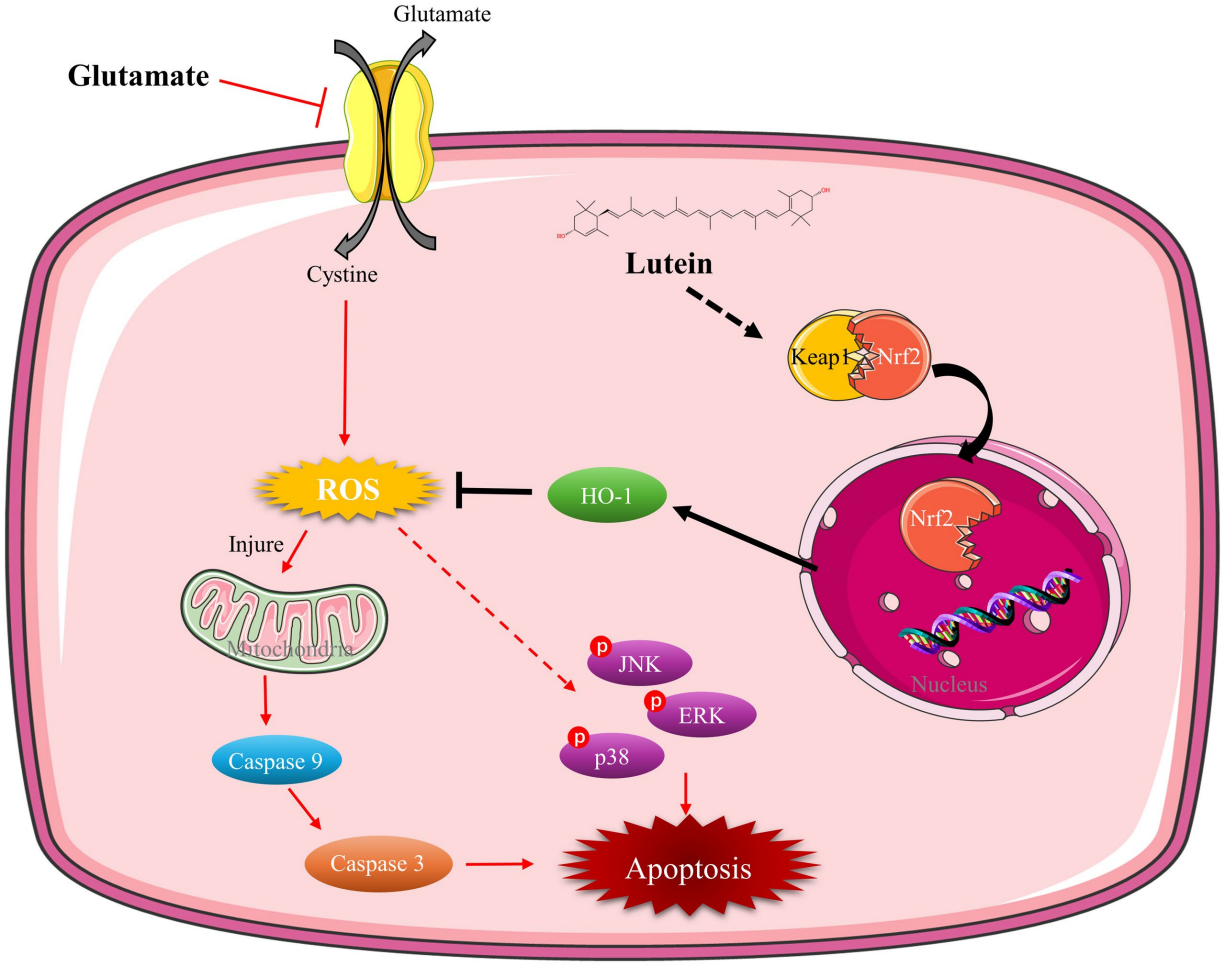
Mechanism of lutein mitigating oxidative stress and apoptosis in glutamate-induced HT22 cells. Lutein facilitates the nuclear translocation of Nrf2, upregulates HO-1 expression, diminishes ROS levels, mitigates mitochondrial damage caused by oxidative stress, inhibits caspase and MAPK activation, and alleviates oxidative stress and apoptosis in HT22 cells.

In summary, our research underscores lutein’s potential as a neuroprotective agent against glutamate-induced oxidative stress and apoptosis in HT22 cells. Lutein demonstrates significant efficacy in reducing cytotoxicity, primarily through its antioxidant properties, which mitigate ROS accumulation within mitochondria and inhibit caspase-mediated apoptosis. Moreover, lutein modulates MAPK pathways and activates Nrf2-mediated antioxidant responses, particularly by upregulating HO-1 expression (Figure 7). These findings highlight lutein’s multifaceted neuroprotective effects and its promise for therapeutic intervention in neurodegenerative diseases characterized by oxidative damage.

## Data availability statement

The original contributions presented in the study are included in the article/Supplementary material, further inquiries can be directed to the corresponding authors.

## Author contributions

ZL: Data curation, Formal analysis, Writing – original draft. ZC: Data curation, Formal analysis, Validation, Writing – review & editing. FC: Data curation, Formal analysis, Validation, Writing – review & editing. BL: Conceptualization, Validation, Writing – review & editing. HJ: Conceptualization, Formal analysis, Funding acquisition, Validation, Writing – original draft, Writing – review & editing.
