# Supplementary material for: Lutein inhibits glutamate-induced apoptosis in HT22 cells via the Nrf2/HO-1 signaling pathway
Source: Front Neurosci. 2024 Aug 13;18:1432969. doi: 10.3389/fnins.2024.1432969 (PMC11347311; doi:10.3389/fnins.2024.1432969)
Supplement: Supplementary file 2 [file Data_Sheet_2.ZIP › WB protein MW markers.pptx]

## Slide 1
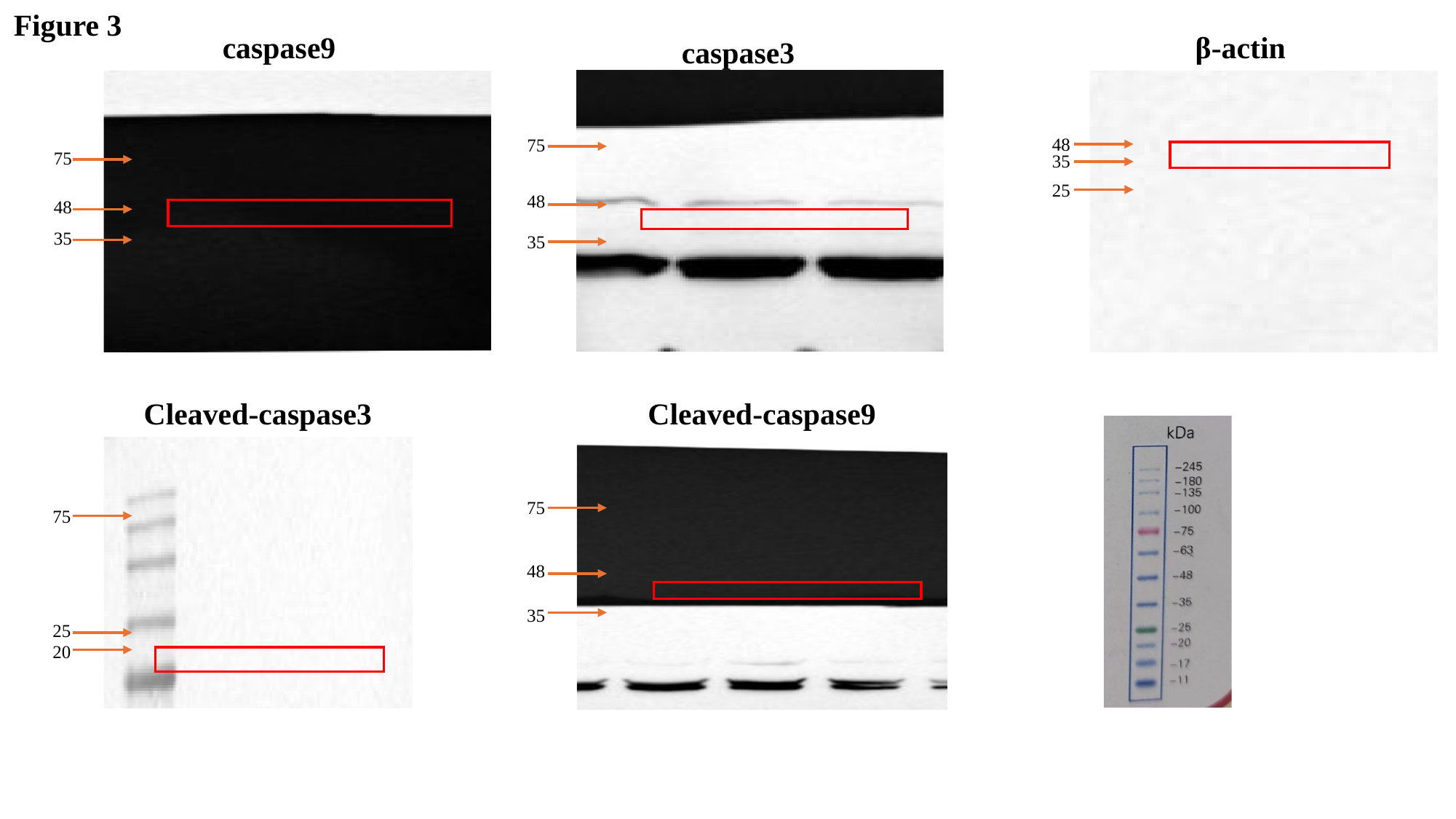

Figure 3
caspase9
β-actin
caspase3
48
75
75
35
25
48
48
35
35
Cleaved-caspase3
Cleaved-caspase9
75
75
48
35
25
20

## Slide 2
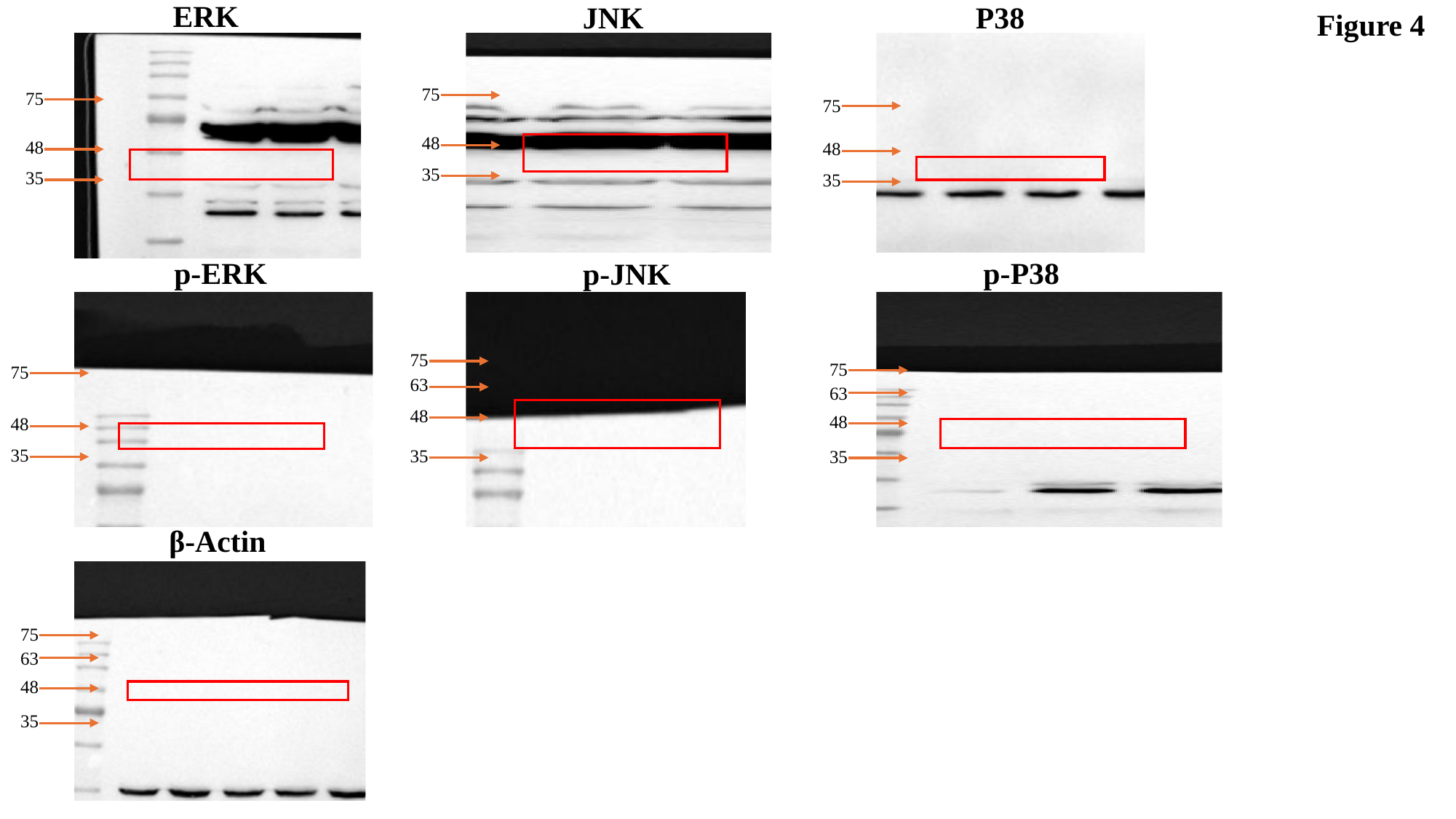

Figure 4
ERK
JNK
P38
75
75
75
48
48
48
35
35
35
p-ERK
p-P38
p-JNK
75
75
75
63
63
48
48
48
35
35
35
β-Actin
75
63
48
35

## Slide 3
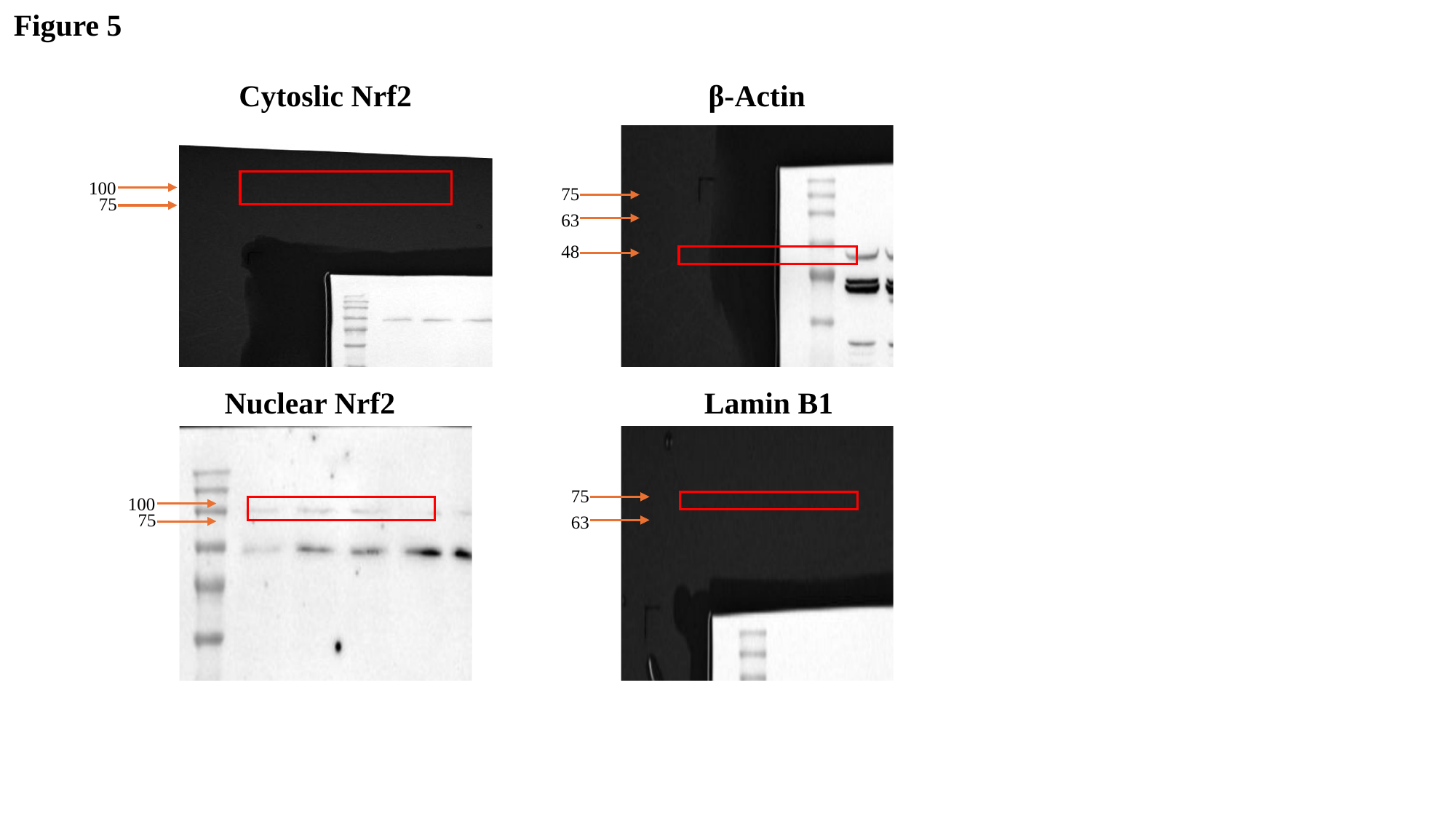

Figure 5
Cytoslic Nrf2
β-Actin
100
75
75
63
48
Lamin B1
Nuclear Nrf2
75
100
75
63

## Slide 4
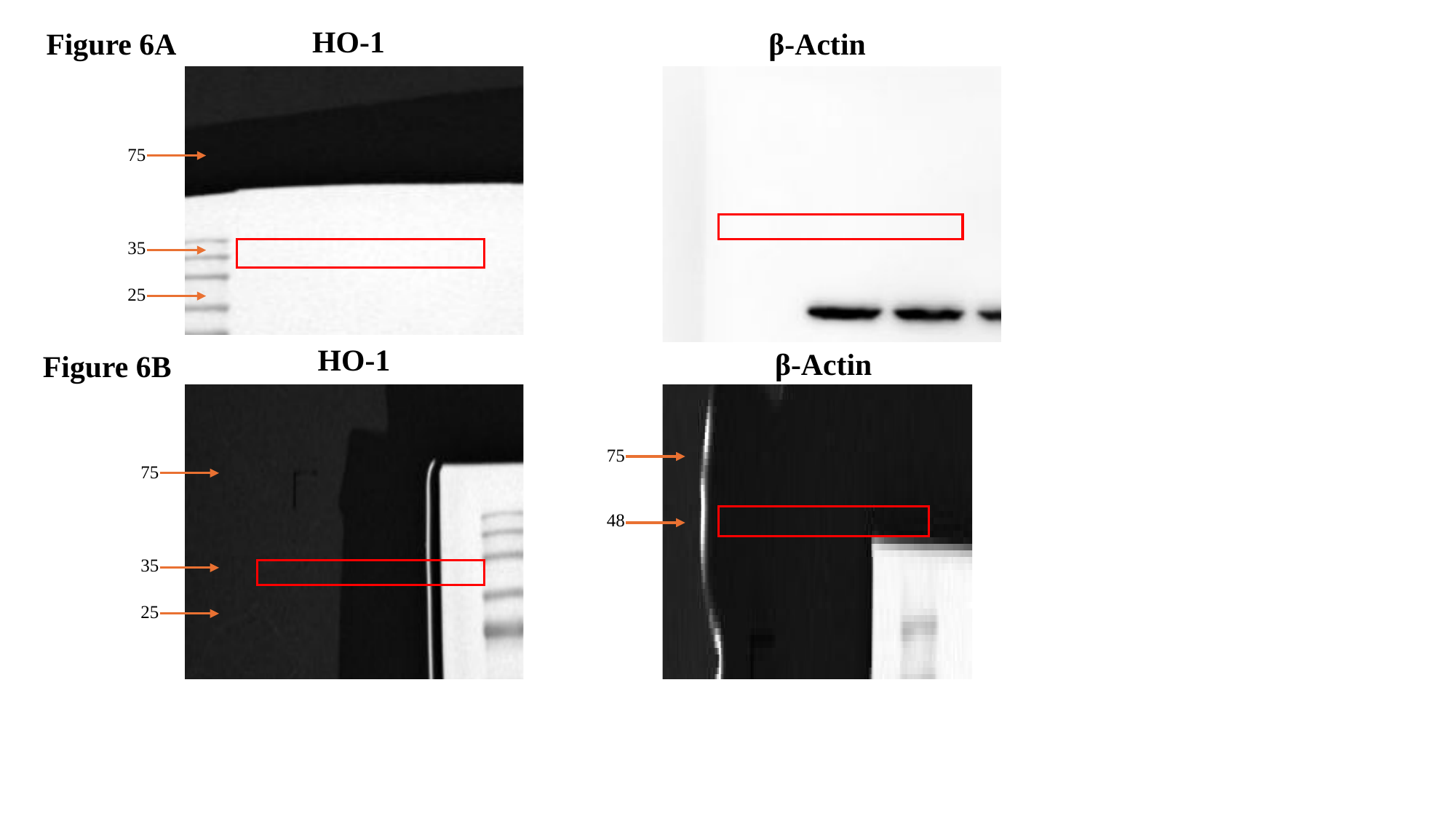

HO-1
Figure 6A
β-Actin
75
35
25
HO-1
β-Actin
Figure 6B
75
75
48
35
25
