# Supplementary figures and images for: Lutein inhibits glutamate-induced apoptosis in HT22 cells via the Nrf2/HO-1 signaling pathway
Source: Front Neurosci. 2024 Aug 13;18:1432969. doi: 10.3389/fnins.2024.1432969 (PMC11347311; doi:10.3389/fnins.2024.1432969)

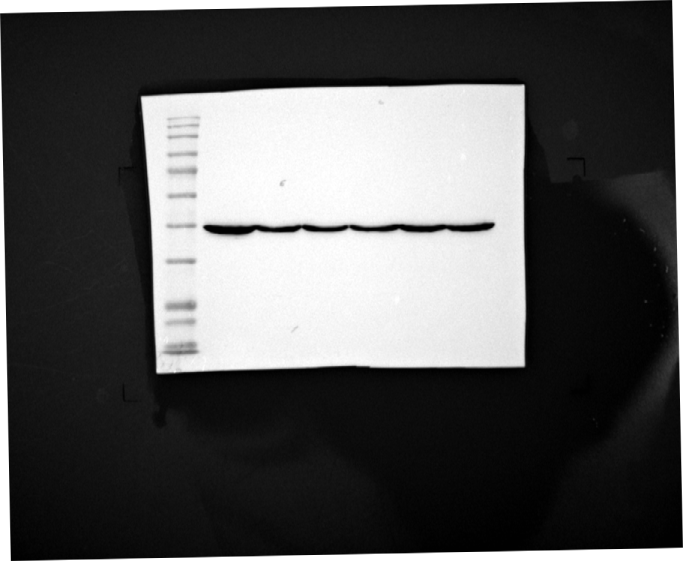

Supplement: Supplementary file 2 [file Data_Sheet_2.ZIP › Fig.3 caspase 9.tif]

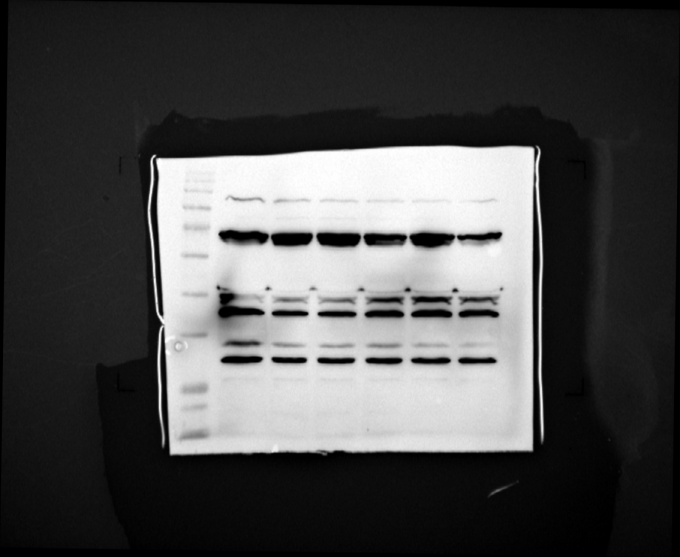

Supplement: Supplementary file 2 [file Data_Sheet_2.ZIP › Fig.3 caspase3.tif]

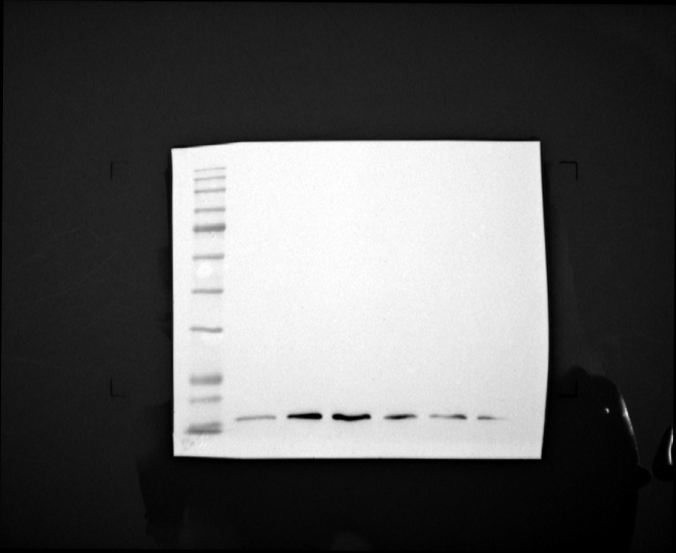

Supplement: Supplementary file 2 [file Data_Sheet_2.ZIP › Fig.3 cleaved-caspase 3.tif]

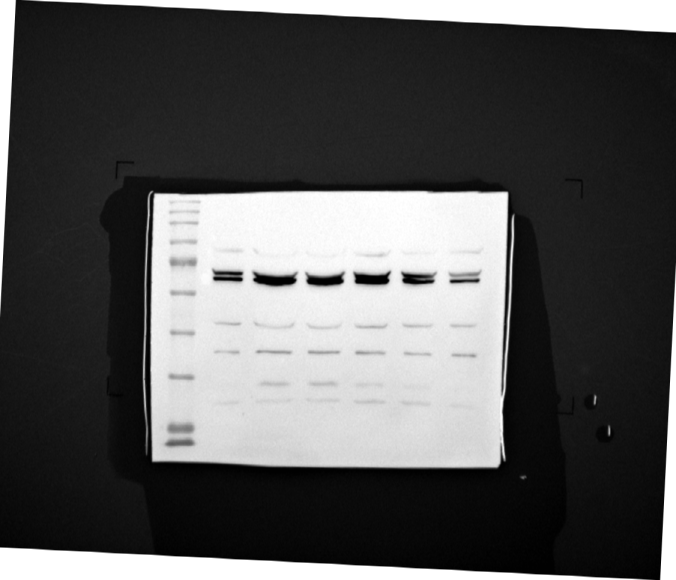

Supplement: Supplementary file 2 [file Data_Sheet_2.ZIP › Fig.3 cleaved-caspase 9.tif]

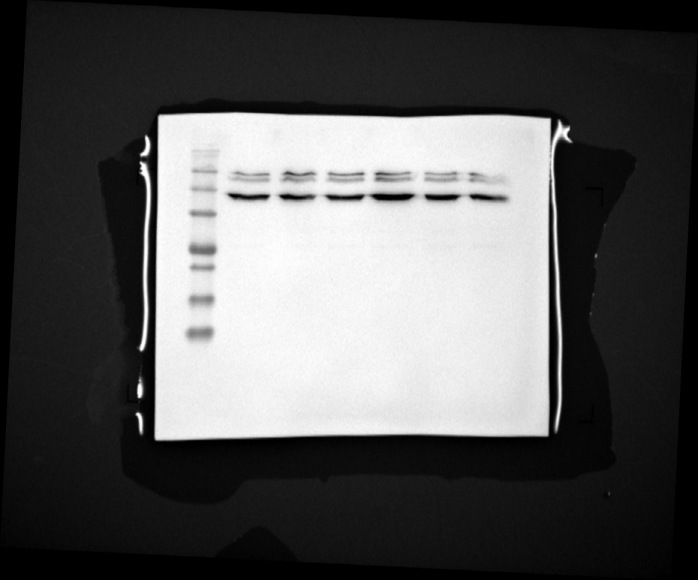

Supplement: Supplementary file 2 [file Data_Sheet_2.ZIP › Fig.3 β-Actin.tif]

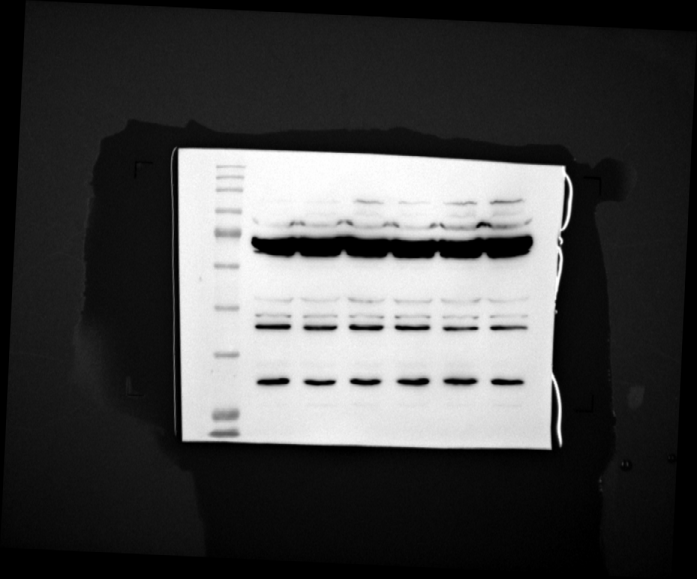

Supplement: Supplementary file 2 [file Data_Sheet_2.ZIP › Fig.4 ERK.tif]

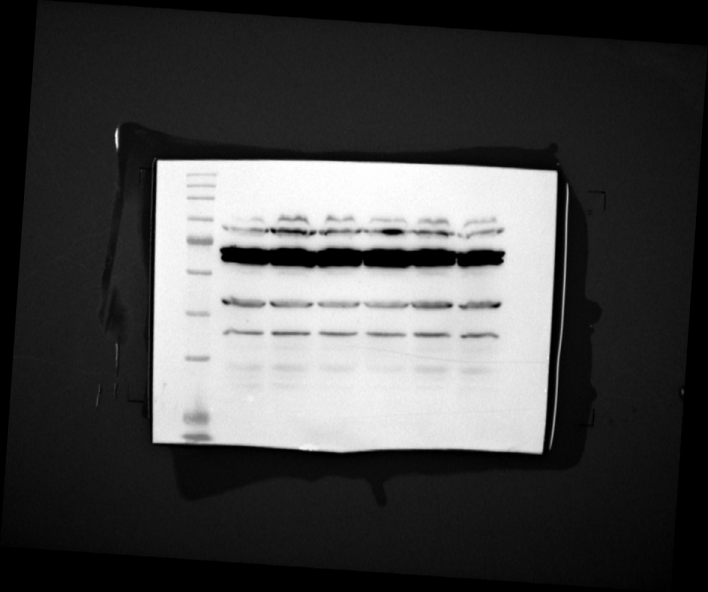

Supplement: Supplementary file 2 [file Data_Sheet_2.ZIP › Fig.4 JNK.tif]

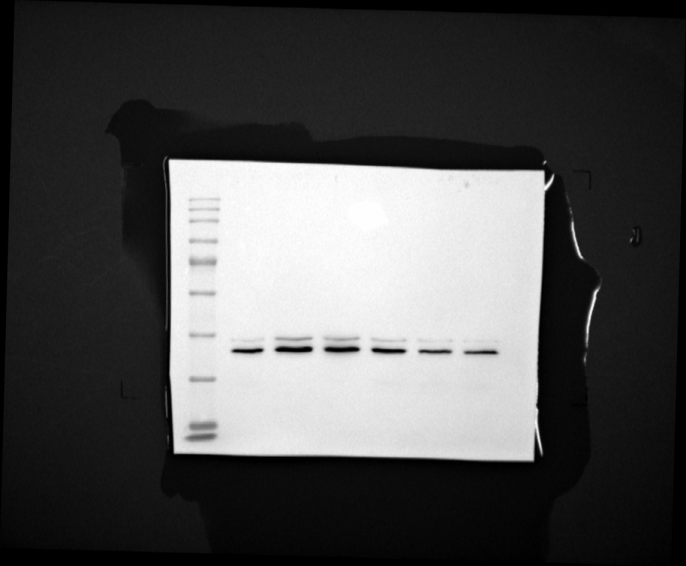

Supplement: Supplementary file 2 [file Data_Sheet_2.ZIP › Fig.4 p-ERK.tif]

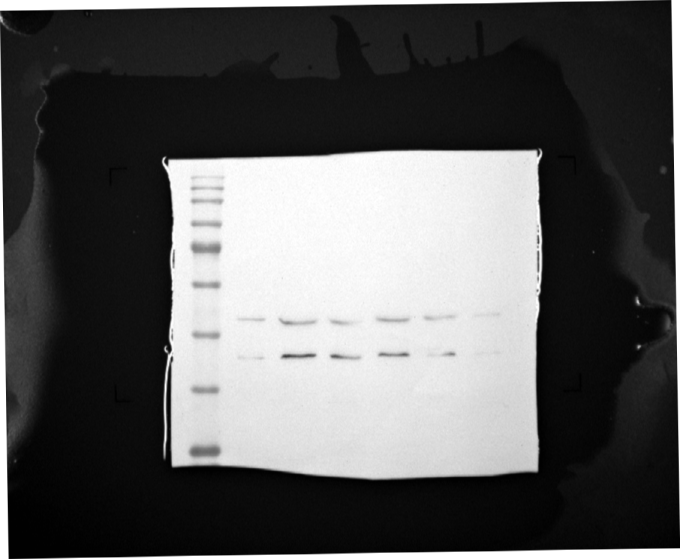

Supplement: Supplementary file 2 [file Data_Sheet_2.ZIP › Fig.4 p-JNK.tif]

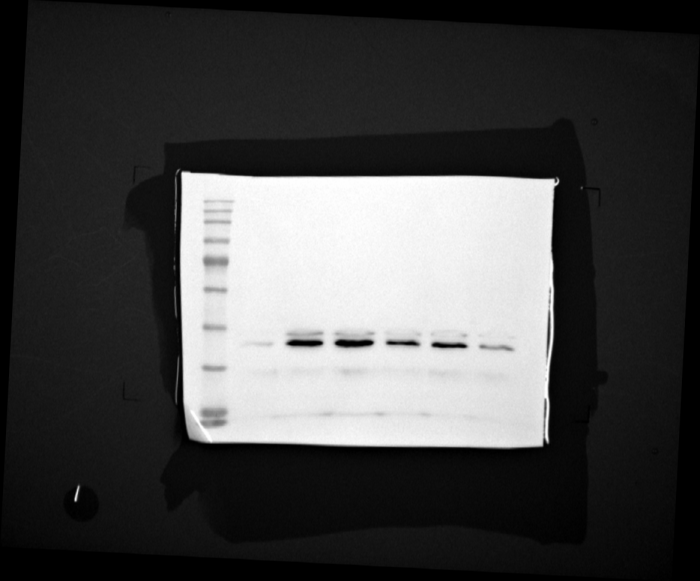

Supplement: Supplementary file 2 [file Data_Sheet_2.ZIP › Fig.4 p-P38.tif]

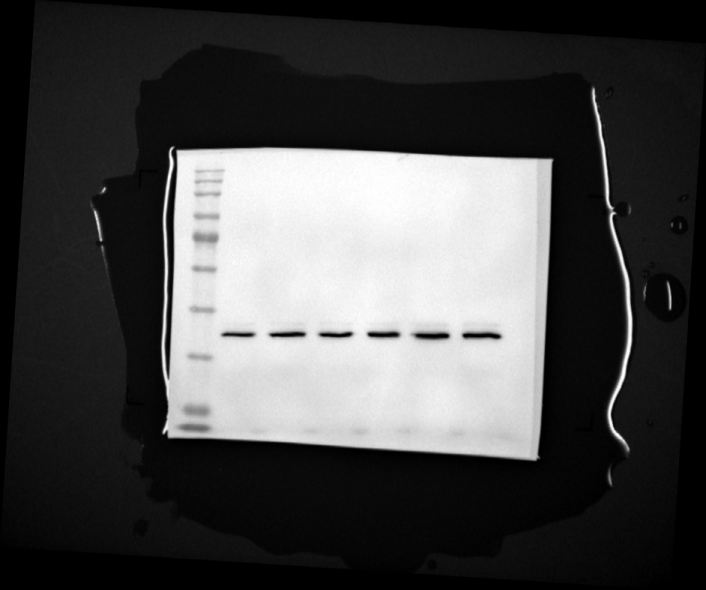

Supplement: Supplementary file 2 [file Data_Sheet_2.ZIP › Fig.4 P38.tif]

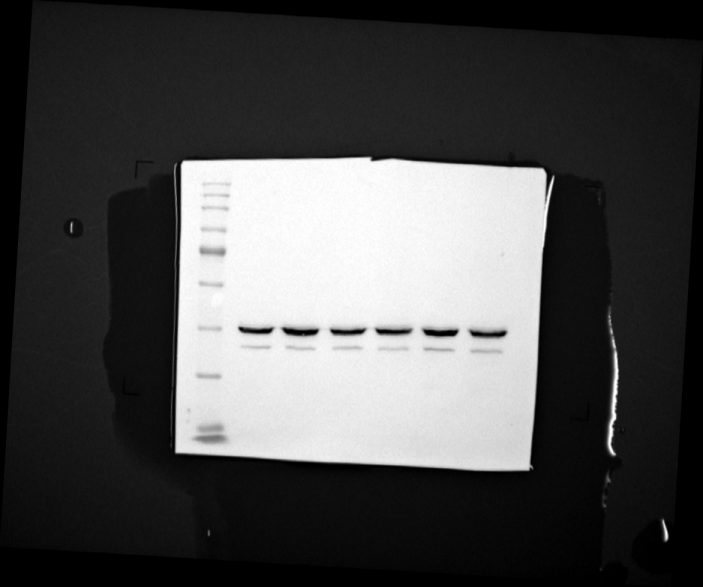

Supplement: Supplementary file 2 [file Data_Sheet_2.ZIP › Fig.4 β-Actin.tif]

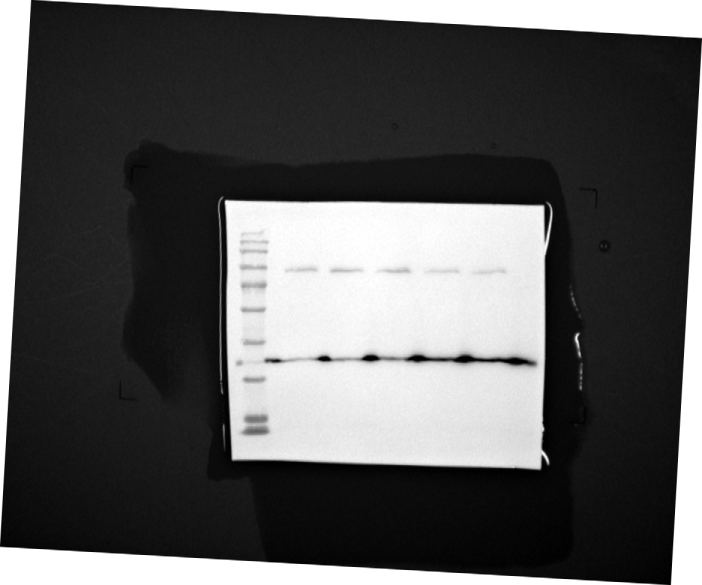

Supplement: Supplementary file 2 [file Data_Sheet_2.ZIP › Fig.5A Cytoslic Nrf2.tif]

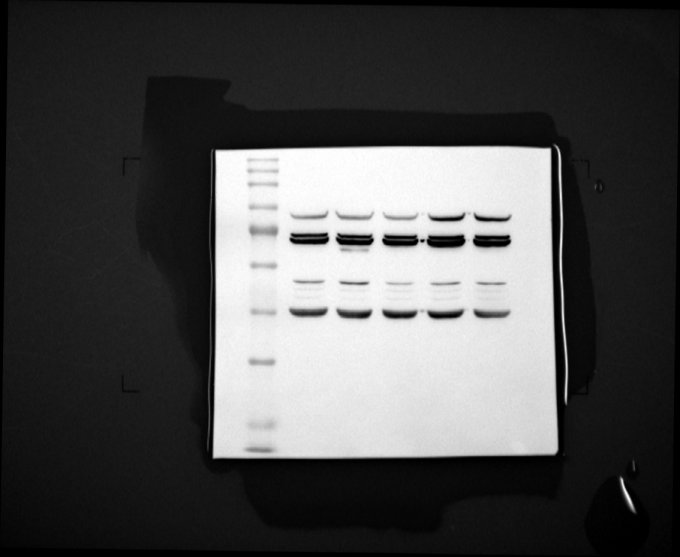

Supplement: Supplementary file 2 [file Data_Sheet_2.ZIP › Fig.5A β-Actin.tif]

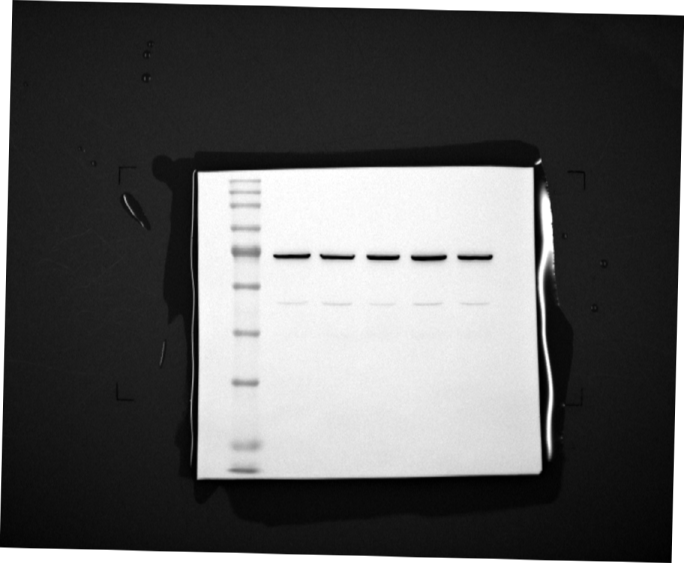

Supplement: Supplementary file 2 [file Data_Sheet_2.ZIP › Fig.5B Lamin B1.tif]

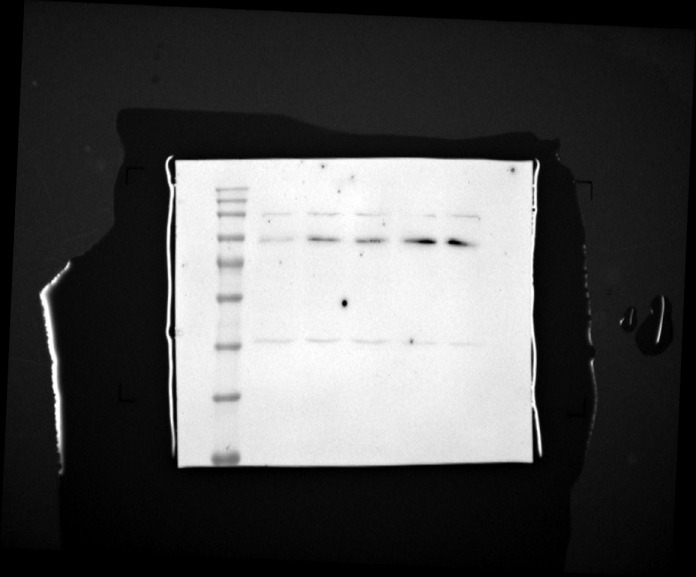

Supplement: Supplementary file 2 [file Data_Sheet_2.ZIP › Fig.5B Nucleus Nrf2.tif]

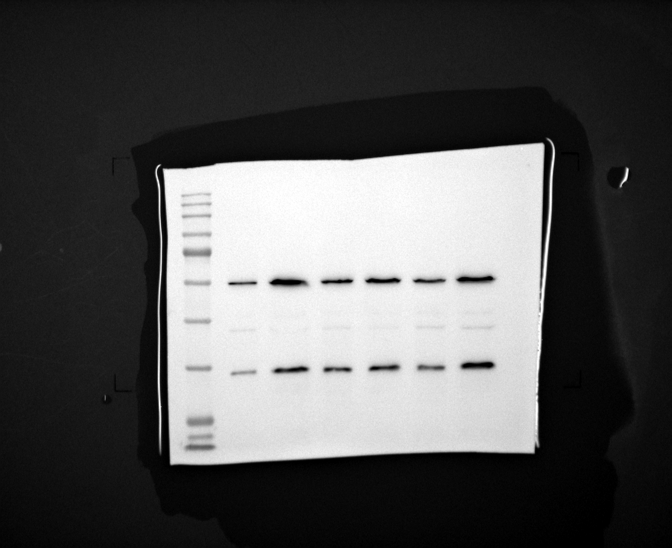

Supplement: Supplementary file 2 [file Data_Sheet_2.ZIP › Fig.6A HO-1.tif]

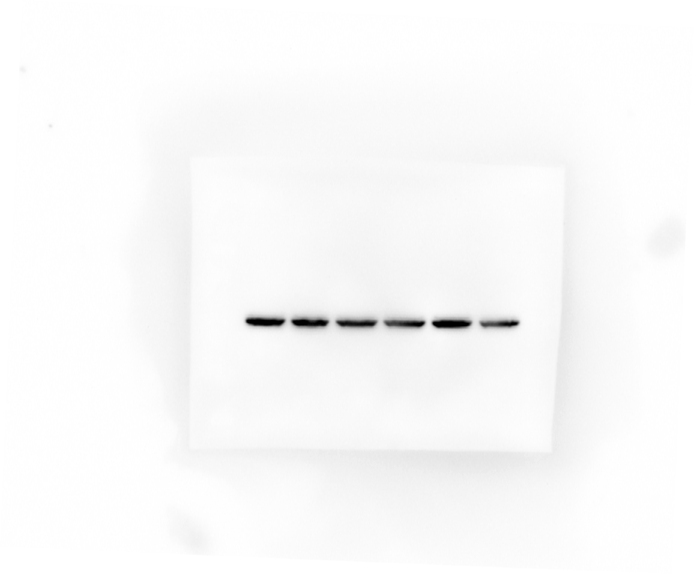

Supplement: Supplementary file 2 [file Data_Sheet_2.ZIP › Fig.6A β-Actin.tif]

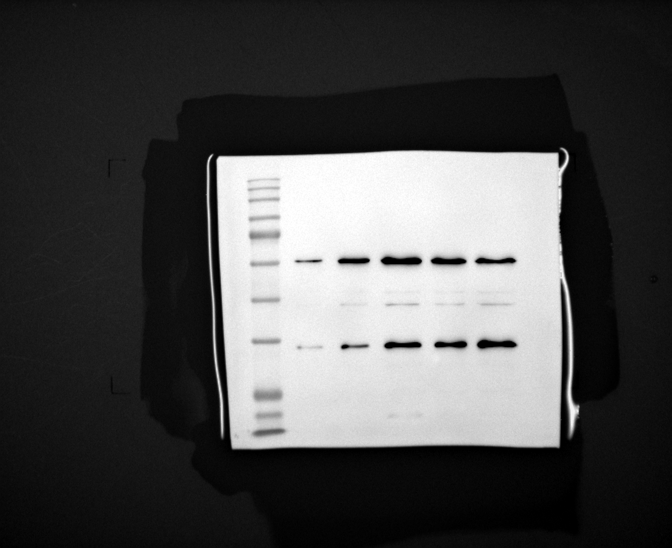

Supplement: Supplementary file 2 [file Data_Sheet_2.ZIP › Fig.6B HO-1.tif]

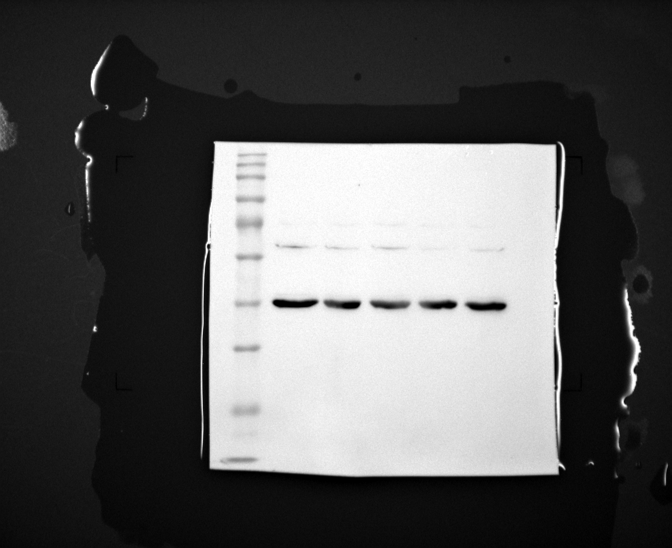

Supplement: Supplementary file 2 [file Data_Sheet_2.ZIP › Fig.6B β-Actin.tif]
